# Tracking and Classification of Head Movement for Augmentative and Alternative Communication Systems

**DOI:** 10.3390/s22020435

**Published:** 2022-01-07

**Authors:** Carlos Wellington P. Gonçalves, Rogério A. Richa, Antonio P. L. Bo

**Affiliations:** 1 SARAH Network of Rehabilitation Hospitals, Brasilia 70000-000, Brazil; 12833@sarah.br; 2 Brazilian National Institute for Digital Convergence, Federal University of Santa Catarina, Florianopolis 88040-900, Brazil; rogerio.richa@scytech.com.br; 3 Electrical Engineering Department, University of Brasilia, Brasilia 70910-900, Brazil

**Keywords:** human–computer interface, human movement analysis, cerebral palsy, hidden Markov model, assistive technology

## Abstract

The use of assistive technologies can mitigate or reduce the challenges faced by individuals with motor disabilities to use computer systems. However, those who feature severe involuntary movements often have fewer options at hand. This work describes an application that can recognize the user’s head using a conventional webcam, track its motion, model the desired functional movement, and recognize it to enable the use of a virtual keyboard. The proposed classifier features a flexible structure and may be personalized for different user need. Experimental results obtained with participants with no neurological disorders have shown that classifiers based on Hidden Markov Models provided similar or better performance than a classifier based on position threshold. However, motion segmentation and interpretation modules were sensitive to involuntary movements featured by participants with cerebral palsy that took part in the study.

## 1. Introduction

The use of computers, tablets, and similar devices is generalized in society. By enabling people to expand their communication possibilities and improve productivity at work, such systems are increasingly part of our daily lives. Nevertheless, to operate conventional keyboards and mouses on computers and laptops, and touchscreen interfaces on smart mobile devices, finger dexterity and range of movement is often a requirement. Hence, millions of individuals worldwide, who lack the ability to control the upper limbs, may not fully experience the functions provided by these devices. The implications of this may be particularly important for children and young adults [1].

Solutions that allow the use of computer systems by a person with disability in order to communicate are referred to as Augmentative and Alternative Communication (AAC). Each individual will adapt himself and use a AAC device based on assessment of cognitive and physical condition. Mechanical switches, switch activated mouses, and sip-and-puff devices are examples of popular systems that deliver input to human–computer interfaces (HCI) that control virtual keyboards and other applications providing means to communication (written or with synthesized speech). In addition to enable use of computer systems, these sensor modalities may also enable control of powered wheelchairs and smart environments, for instance [2]. Nonetheless, despite their ease-of-use, robustness, and reduced cost, mechanical switches present limitations, such as the need to re-position the sensor if the body posture changes, and the requirement of complex mounting structures fixed to the wheelchair [2].

For those conditions in which the individual presents only head and facial functional movements, systems based on eye and head tracking are valuable alternatives, particularly if no markers are required. Among the markerless solutions already available for the user, some require specialized hardware in order to track eye movement, such the Tobii eye tracking systems (Tobii, Sweden). However, despite positive evaluation from parents [3] in a pilot study, they require the user to maintain firm sitting position and avoid gross head movements for suitable operation. Hence, for some individuals, such as those with dystonia, spasticity, or pathological tremor, these solutions are not functional. Furthermore, since most computer systems currently feature onboard cameras and thus enabling the design of an AAC device with no additional hardware, such AAC systems are costly when compared to other alternatives.

Concerning markerless systems that employ a conventional webcam, software is also available that enables head tracking and estimating the corresponding cursor movement. Indeed, systems that rely on cameras to enable HCI using head tracking feature additional advantages, mostly related to the wide availability of cameras in modern devices. However, they share similar limitations to eye tracking systems. Hence, solutions, such as the Headmouse (Universitat de Lleida, Spain) [4] require fine control of head movements. These limitations may likely persist even if novel sensing technologies (e.g., Kinect, as in [5]) or computer vision tracking tools (e.g., [6,7,8]) are used with this purpose.

Based on the literature, for users who feature involuntary movements or disability that interfere with head movement, there is no viable alternative to the best of authors knowledge of computer vision based systems enabling effective AAC. In this scenario, in this work we propose applying head tracking using computer vision alongside gesture recognition methods for enabling robust detection of specific head movements that are then used for AAC. In particular, two user intent detection approaches are proposed and compared experimentally in this paper: while one relies on simple position thresholds, the other is based on Markov modeling to characterize and recognize appropriate movements. Experimental studies have been conducted in two groups, namely participants with disabilities and participants presenting no neurological impairments. The experimental protocol has been designed to enable evaluating the overall feasibility of the method, as well as its performance, particularly in terms of minimizing false positives.

This paper is organized as follows. Section 2 presents the method, including information on participants and experimental protocol, as well as a detailed description of the proposed HCI system based on computer vision and intention classification. Experimental evaluation is presented in Section 3, including data from individuals with cerebral palsy (CP) and participants with no neurological disorder. These results are discussed in Section 4. Finally, we draw conclusions and discuss future works in Section 5.

## 2. Materials and Methods

The AAC system proposed in this paper relies on the coordinated performance of distinct methods, which are described in this Section. Information regarding recruitment and experimental protocol are also provided here.

### 2.1. Subjects and Protocol

Six subjects in total were recruited for this study, four participants with no neurological disorder (group A, three males) and two participants with cerebral palsy (group B, two males). The research was approved by the Ethics Committee at the SARAH Network of Rehabilitation Hospitals and registered in Brazilian National Committee in Research Ethics (CONEP) with CAAE number 15055513.6.0000.0022, in accordance with the Helsinki Declaration. All volunteers signed an informed consent form.

Participants from group B who participated in the study presented heterogeneous features in terms of movement disorder. Although participant B1 featured involuntary movement mostly described as choreodystonia, participant B2 presented mostly choreoathetosis. Both participants had previous experience in using mechanical switches for AAC. Group B participants were explicitly recruited to evaluate system performance when users present intense dystonia. Group A participants were selected to enable assessing system performance when users present no involuntary movement, a condition that represents several targeted clinical populations.

The study consisted of trials in which participants were given explanatory information on the protocol and then seated comfortably in front of a computer screen with a webcam (640×480 pixels resolution). A mechanical switch was positioned next to the participant’s head, in order to control a virtual keyboard with scanning control scheme. Each participant then performed head movements that triggered the mechanical switch in order to write a predefined sentence. Sentences were defined such that the expected duration to type it was similar for participants in groups A and B. Participants were instructed to focus on head movements, particularly side-bending and flexion, but trunk movements were also allowed. Group A participants executed complementary movements guided by a orange sphere randomly placed next to the participant’s head by the researcher conducting the experiment. This additional step was included to enable evaluating head detection and tracking performance in the presence of head motion that should not generate writing (i.e., invalid movements). All movement was registered, as well the reference valid movements obtained from the mechanical switch activation, for further analyzing the proposed HCI system.

### 2.2. Head Detection and Tracking

Face detection is the first stage within the head tracking method employed in this work. Its implementation is obtained using a Viola–Jones classifier trained to vertical face recognition [9].

Among the candidates for the user’s face, only the largest rectangle is analyzed. We assume that the user is positioned in front of the webcam and thus he is the person closer to the camera. This approach allows multiple individuals to be present in the image, with no compromise to function.

At the end of this stage, both the rectangle dimensions that delimits the user’s face, $s_{h0}=\left(w_{h0},h_{h0}\right)$, and the corresponding position of this rectangle, $p_{h0}=\left(x_{h0},y_{h0}\right)$, are obtained. For each different position of the user in the image, either in the plane (x,y) or in relation to distance from the camera, different $s_{h0}$ and $p_{h0}$ may be obtained. However, this variability is unsuitable for tracking. For this reason, two additional operation are performed on the reference image: scaling and centering. The scale factor is calculated to generate a region of interest whose width is three times $w_{h0}$. Both operations are then performed for every frame of the acquired video.

Head tracking is then implemented using primarily the mean shift algorithm, particularly employing the posterior probability measure as a similarity measure [10] modified to work with colored images coded in HSV domain. Among the implementation choices in this stage, the rectangle dimensions are reduced in 20% to partially remove the user’s hair and the search region is defined as 50% larger than the original scaled rectangle.

**Figure 1:**
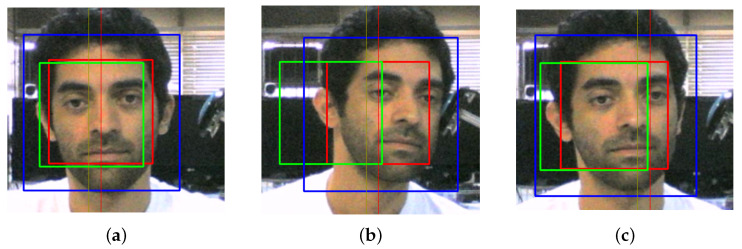
Illustration of head tracking and threshold-based movement classification. The green rectangle represents the reference position, denoted by $s_{h0}$ and $p_{h0}$, while the red rectangle illustrates the current estimated position. (**a**) illustrates the initial position, (**b**) the definition of a position threshold $x_{t}$ (yellow line) based on $\max\left(x_{h}\right)$ (red line), and (**c**) the user moving his head to control the virtual keyboard.

Furthermore, since this estimate will often be affected by noise and other error sources related to lighting and inherent limitations of the method, a Kalman filter is used for additional filtering. In this case, a constant-velocity model is employed, and the corresponding covariance matrices were selected empirically to improve motion segmentation performance. Figure 1 illustrates both reference detected position and estimated tracked position.

### 2.3. Motion Segmentation and Classification

Motion segmentation is used with the goal of reducing the amount of data that require classification and also to determine the segments candidates that represent functional movements. Our approach was conceived based on the methods proposed in [11,12].

Considering head velocity estimates provided by the Kalman filter, segmentation is performed based on a zero velocity cross approach, where the following quantity is calculated to combine the effects of *x* and *y* trajectories:

(1) $$\begin{array}{c} \epsilon ={\dot{x}}_{h}^{2}+{\dot{y}}_{h}^{2}. \end{array}$$

In this work, only reaching movements followed by return to the reference position are considered as potential input to the system. Hence, we have implemented a finite-state machine to detect, based on ϵ, one peak detected between two valleys, which then represents the segmented movement.

Two different methods were used to classify a movement as functional (also referred to as valid in this context). Although one method is based on a *x*-axis position threshold, the other is based on Markov modeling, which potentially enables more complex and accurate modeling of the functional movement.

For implementing the first method, i.e., detecting the functional reaching movement based on a horizontal threshold $x_{t}$, motion segmentation is not required. $x_{t}$, which is defined in relation to the reference head position $x_{h0}$, is calculated during an initial calibration phase based on maximum displacements when performing the reaching movement, $\max\left(x_{h}\right)$. In this works, we have used the average when performing 10 reaching movements. The threshold $x_{t}$ is defined as 20 pixels closer to the reference position, as illustrated in Figure 1. Since the user may perform reaching movements towards any direction, both positive and negative $x_{t}$ are possible.

The second method is based on the understanding that the reaching movements are composed of stages. The approach firstly involves obtaining the Hidden Markov Model (HMM) using segments representing valid movements. Based on these segments, clustering using k-means was employed to obtain the statistics of each stage that compose the functional movement sequence. Four-state HMMs were chosen to represent the desired movements, and hence the number of possible clusters was set to four. Next, the probability density functions of each cluster were used as an initial estimate of the state observation matrix B. Finally, the corresponding transition matrix A and the initial distribution π are estimated using the Baum-Welch algorithm. This procedure was applied for three different feature vectors evaluated in this work: head position (HMM-P), velocity (HMM-V), or both (HMM-PV).

Identifying valid movements using the HMM-based method is primarily accomplished using the log-likelihood, $l_{s}$. Furthermore, early trials indicated influence of the segment length, $n_{s}$. This joint measure $\psi ={\left[l_{s}\;n_{s}\right]}^{T}$ is the basis for detecting valid segments. The normal distribution of ψ obtained using valid segments is calculated, and the corresponding Mahalanobis distance to ψ given by a measured segment is used for classification.

### 2.4. Data Analysis

The main outcome measure in this work was obtained using the receiver operating characteristic curve, or ROC curve. In a ROC curve, the corresponding threshold is varied to generate the corresponding true positive rate (TPR) against the false positive rate (FPR), which were both calculated based on the ground truth (i.e., activation of the mechanical switch). In the HMM-based method, the threshold refers to confidence level employed on the null hypothesis testing using the Mahalanobis distance. Finally, the area under the curve (AUC) is used to provide a quantitative measure of each classifier.

Furthermore, regarding the HMM-based method, evaluation of preliminary results generated in this study was performed using 3-fold cross-validation, where a third of collected data were used for validation at each iteration.

## 3. Results

Table 1 lists the main result in this proof-of-concept study. The AUC calculated for every participant using both movement intent detection methods are listed. For the HMM-based method, results obtained for each evaluated feature vector are included.

**Figure 2:**
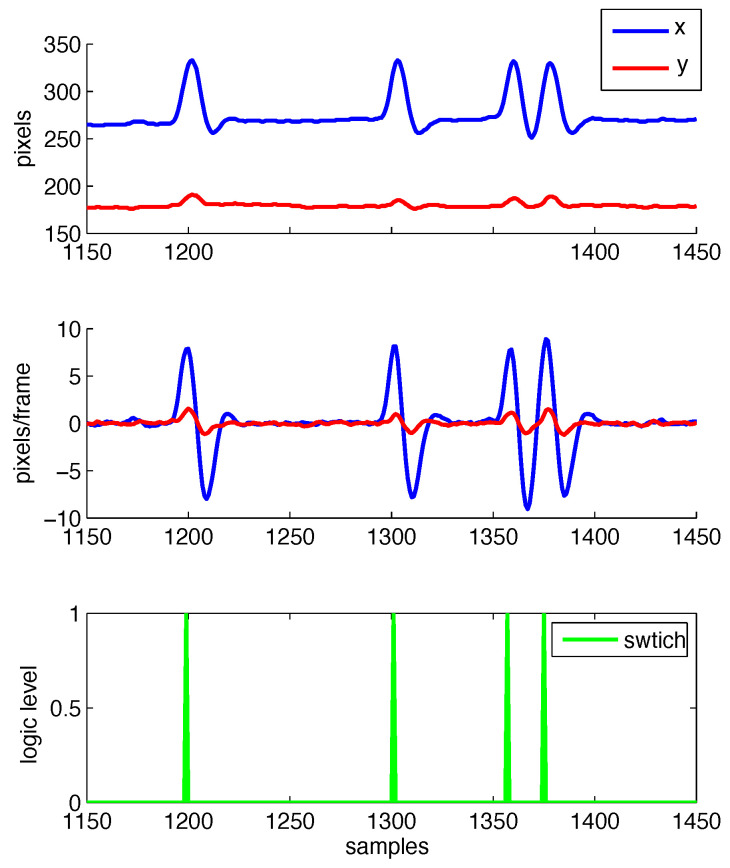
Illustration of head tracking performance, both in terms of position (**top**) and velocity (**middle**). Corresponding activation of mechanical switch is also depicted (**bottom**).

**Figure 3:**
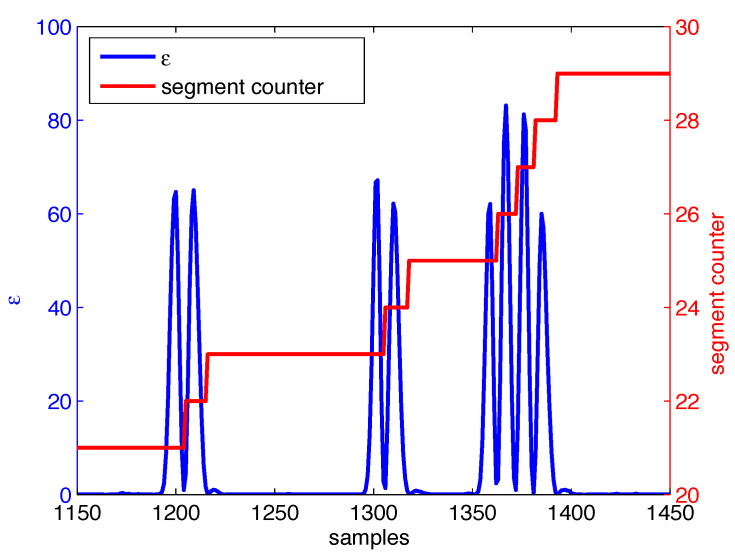
Example of motion segmentation based on ϵ, including corresponding segment counter.

**Figure 4:**
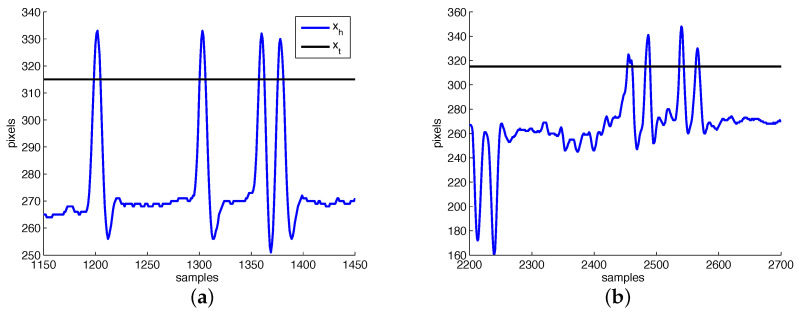
Sample classification results when using the threshold-based method. In (**a**) four successive reaching movement are successfully detected, while in (**b**) false positives due to involuntary movements are depicted.

**Figure 5:**
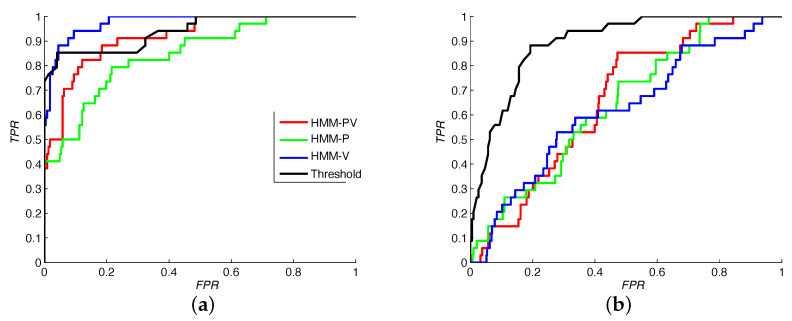
ROC curves obtained for participants (**a**) A2 and (**b**) B1. All classifiers are compared using 3-fold cross-validation.

Additionally, Figure 2, Figure 3, Figure 4 and Figure 5 illustrate intermediate signals and ROC curves generated with the experimental data. In particular, Figure 2 serves to illustrate the performance obtained using the head tracking method proposed in this work.

Figure 3 and Figure 4 depict, respectively, the motion segmentation method and the threshold-based classification method. In Figure 3, the detection of each segment (described in Section 2.3) is illustrated by the segment counter, which is incremented once the finite-state machine detects a peak between two valleys in ϵ. Regarding the threshold-based method, Figure 4a illustrates cases where the threshold-based method performs satisfactorily, while in Figure 4b one false positive occurs (first positive movement) among a total of six detected segments.

Considering the Table 1 and Figure 5, while the performance between HMM-based and threshold-based classifiers may seem similar at first, in some cases the HMM (and in particular HMM-PV and HMM-V) performance in terms of low FPR stands out an important feature.

Table 1 also shows similar AUC was obtained for participants A1, A2, and A3. For participant A3, the lower performance by HMM-P possibly is due to the lack of velocity-dependent features. The corresponding transition matrices have shown that HMM-PV and HMM-V often generate classifiers with higher capacity of generalization.

For users A4, B1, and B2, issues were observed in the generation of training segments with a substantial number of samples. For A4 the main problem was possibly the proximity of the mechanical switch to the user’s face. For B1 and B2, the presence of involuntary movements prevented the acquisition of longer segments, deteriorating the performance of the HMM-based method for all features (as illustrated in Figure 5b. Nevertheless, it may also be observed that the performance of the threshold-based classifier also deteriorated due to variations in range of valid motions. For participants in group B, this often occurred because the switch was often pushed with intense force, shifting its position. Since participant B2 featured a more controlled movement, a better performance was obtained for all classifiers when compared to participant B1.

## 4. Discussion

To the best of the authors knowledge, this paper presents a first attempt to implement an AAC system using head tracking and user intent recognition based on HMM. Nevertheless, while the proof-of-concept experiments helped us to evaluate the overall usability of the system, some of the potential limitations became evident.

The group of people who activate mechanical switches employing head movements to use a computer is the target population in this study, particularly the subgroup that also features involuntary head movements. This is also an important advantage of the approach proposed in this paper, since little training is expected to use the system, since similar movements as those required to operate the switch are employed as input in this work. Another fundamental feature of our solution regards the low technical requirements. Indeed, the two hardware elements required in the system are a simple webcam to acquire images of the user and a basic computer to run the software application that interprets the head movements to enable control of virtual keyboard.

One specific challenge in systems that enable control of virtual keyboards, particularly when sweep scanning mode is used, refers to false positives (e.g., selecting a wrong character when using a virtual keyboard). Indeed, often a high number of additional steps need to be performed by the user in virtual keyboards or general AAC software whenever a false positive occurs. This issue becomes a larger concern if we consider that voluntary activation often demands a high level of user concentration, as well as being physically demanding. These are the fundamental reasons of our concern to reduce the number of false positives generated by the system.

In this work, evaluation of performance was based on tests where participants were instructed to type predefined sentences using mechanical switches activated by their head movements. Video was also recorded, which enabled comparison between the various methods based on computer vision proposed in this work with the assistive device that is commonly used by this population (i.e., mechanical switches). Nevertheless, it also means that we have not formally evaluated each individual component of our algorithm, such as the tracking and segmentation routines. Although preliminary data indicating satisfactory tracking performance is presented in Section 3, analysis of segmentation performance is indirect, based on final classification results.

Regarding the classification in this work, we have proposed two different user intention detection systems. The first is based on a head position threshold, while the second is based on Markov models. Both systems are trained from movements labeled as valid, which are identified when the user is writing a sentence using a mechanical switch and an on-screen keyboard using sweep scanning mode. The threshold-based method draws clear inspiration on mechanical switches. The modeling of functional movement is performed based on the expected range of motion alone. However, involuntary movements of the same range are treated in the same way (e.g., Figure 4b), effectively limiting the usability of the method for certain populations who present levels of involuntary movement that overlap the assigned threshold.

Although the threshold-based method may rely on the horizontal estimate of head position only, this is not the same for the HMM-based method. Clearly, computer vision tracking systems can provide further information in addition to the *x*-axis position of an object. Using the Meanshift algorithm based on the PPM similarity measure, we estimated the user head position, estimating velocity and position in two dimensions. Although other techniques could be used to enable head tracking, often providing other variables, such as head orientation, in this work we hypothesized that a simple but robust tracking would provide sufficient performance.

The application of a Markov chain proved to be extremely flexible, while simultaneously requiring few initialization parameters. The main parameters defined were the number of states, i.e., four, and the use of observable data sampled from a continuous distribution. The model training employs data from valid movements alone, and the classification of a given movement by the model is performed using a continuous variable that represents the probability of this movement being represented by the chain.

For participants who do not experience involuntary movement (i.e., group A), the performance of the HMM classifiers surpassed in some cases the threshold-based classifier. That may have occurred due to the incorporation of an actual model of the valid movement, which is the basis for disregarding involuntary movements of similar amplitude. However, participants from group A who featured very small functional movements generated valid segments with few samples (e.g., A4). In these cases, the resulting model often presented more than one state that do not allow transition to others states.

For participants who present involuntary movements (i.e., group B), the training of the Markov model was affected by limitations in the segmentation algorithm employed in this work. Functional movements were split due to the sudden change in speed, such as depicted in Figure 4, generating once again segments of valid movement with few samples. The higher number of invalid segments generated by involuntary movements finally increased the number of false positives obtained for these data. We can infer that, based on these results, this use of this specific classifier for this population is unfeasible.

Based on these factors, one possible conclusion is that in this work we could not fully assess the hypothesis that the higher-dimensional representation of movement in HMM-based classifiers (i.e., displacement and speed in *x* and *y*, in comparison to displacement in *x* only) generates improved AAC systems. Involuntary movement not only compromised the performance of the threshold-based method, but also the HMM-based method, demonstrating how the modeling adopted in this work may have been insufficient to detect the volitional movement. Indeed, users with disabilities featured head movement patterns that were barely perceptible to the naked eye, which also lead us to infer a higher-dimensional model might be required to provide correct classification.

In order to minimize the issues related to the segmentation performance, we understand that solutions that allow the creation of segments with a larger number of samples may enhance the classification accuracy of HMM-based methods, particularly decreasing the rate of false positives. Nevertheless, given the issues discussed here, further methods to take into account the effect involuntary movements should be considered to enable robust replacement of AAC based on mechanical switches, particularly for those individuals with severe movement disorders. One of such methods would involve modeling the involuntary movement itself, such as in [13,14], while another approach would involve providing a more comprehensive framework for defining head movements to operate the HCI, such as in [15].

Lastly, while a writing task was used in this work to evaluate the feasibility of the proposed HCI system, in our research group we are interested in evaluating similar methods in other AT scenarios. Some examples applications involve human–robot interaction [16,17] and wearable systems [18].

## 5. Conclusions

The availability of easy-to-use AAC systems based on tools from computer vision may produce a high impact on the quality of life of individual with severe motor disabilities. Nevertheless, alternatives are scarce for those who feature intense dystonia, a common manifestation on children and young adults with CP. In this work, we have presented a self-contained method developed using open-source libraries that enables tracking head movements and detecting user’s intent, which may then be used to control a virtual keyboard or other system. An experimental proof-of-concept study was conducted involving participants with CP and subjects with no neurological disorders. The obtained results have indicated satisfactory outcome for both techniques compared in this paper, whereas the performance was reduced whenever intense involuntary movement occurred.

## Figures and Tables

**Table 1:** AUC for each participant and every user intent detection method.

Participant	HMM-PV	HMM-P	HMM-V	Position Threshold
A1	0.997±0.008	0.997±0.007	0.997±0.008	0.964±0.030
A2	0.916±0.061	0.846±0.132	0.976±0.03	0.94±0.146
A3	0.981±0.055	0.829±0.281	0.982±0.017	0.995±0.021
A4	0.866±0.198	0.817±0.236	0.853±0.18	0.926±0.146
B1	0.657±0.096	0.642±0.315	0.62±0.348	0.892±0.163
B2	0.772±0.389	0.78±0.425	0.779±0.599	0.947±0.016

## Data Availability

Not applicable.

## Abbreviations

The following abbreviations are used in this manuscript:

AAC	Augmentative and Alternative Communication
AUC	Area Under the Curve
AT	Assistive Technology
CP	Cerebral Palsy
FPR	False Positive Rate
HCI	Human–Computer Interface
HMM	Hidden Markov Model
ROC	Receiver Operating Characteristic
TPR	True Positive Rate
